# Kairomonal Effect of Aphid Alarm Pheromones and Analogs on the Parasitoid *Diaeretiella rapae*

**DOI:** 10.3390/insects13111055

**Published:** 2022-11-15

**Authors:** Yaoguo Qin, Shangyang Zhang, Zhengxi Li

**Affiliations:** Department of Entomology and MOA Key Laboratory for Monitoring and Environment-Friendly Control of Crop Pests, College of Plant Protection, China Agricultural University, Beijing 100193, China

**Keywords:** aphid alarm pheromone, analog, blend, kairomone, *Diaeretiella rapae*

## Abstract

**Simple Summary:**

Aphid alarm pheromones, as important semiochemicals, not only mediate the behavioral response of aphids, but can also act as kairomones to attract their natural enemies. The sesquiterpene (E)-β-farnesene (EβF), the major alarm pheromone component of most aphid species, has been shown to have a kairomonal effect on the predators of aphids, but other alarm pheromone components, especially the monoterpenes and analogs, are rarely investigated. In this study, we examined the kairomonal effects of four alarm pheromone components and two EβF analogs on the aphid parasitoid *Diaeretiella rapae*. We found that the blend of aphid alarm pheromone components is more preferred by the parasitoid than are the individual components. Even if individual pheromone components showed no kairomonal activity at lower concentrations, the blending of these components elicited a response. This study contributes to our understanding of the mechanisms involved in the regulation of parasitoid behaviors by kairomones and provides a promising opportunity for designing kairomones for the natural enemies of aphids to mediate aphid populations in the field.

**Abstract:**

Aphid alarm pheromones, as important semiochemicals, not only mediate behavioral response of aphids, but can also act as kairomones to attract their natural enemies. The sesquiterpene (E)-β-farnesene (EβF), the major alarm pheromone component of most aphid species, has been shown to have a kairomonal effect on the predators of aphids, but other alarm pheromone components, especially the monoterpenes and analogs, are rarely investigated. Here, two EβF analogs were successfully synthesized via the nucleophilic substitution reaction, and we then examined the kairomonal effects of four alarm pheromone components and two EβF analogs on the aphid parasitoid, *Diaeretiella rapae*. In olfactory bioassays, *D. rapae* females generally showed no significant behavioral response to these alarm pheromone components and analogs under low concentrations (0.1 μg/μL). Nevertheless, their olfactory response to these compounds gradually enhanced with increasing concentrations. Among the four pheromone components, EβF showed the highest attractive activity, but the parasitoid preferred blends over single compounds. Moreover, the response time decreased as the concentration increased. We confirmed the kairomonal effect of monoterpene alarm pheromone components and their blends, in addition to EβF, on the natural enemies of aphids. This is the first report that the blend of alarm pheromone components and their analogs has a stronger kairomonal effect than do the single components on the natural enemies of aphids. This study contributes to our understanding of the mechanisms involved in the regulation of parasitoid behaviors by kairomones and provides a promising opportunity for designing kairomones for the aphid parasitoid to mediate aphid populations in the field.

## 1. Introduction

Insect predators and parasitoids use semiochemicals such as plant volatiles, insect sex pheromones, and alarm pheromones to locate plant hosts, find mates, and avoid natural enemies [1,2,3]. Aphids occur throughout the world, causing serious damage to agricultural and horticultural crops because of their large populations, rapid breeding, and wide host range [4,5]. Aphid alarm pheromones are sticky droplets secreted from the cornicles of aphids when attacked or disturbed by parasitoids or predators, causing nearby conspecifics to disperse or escape [6,7]. The sesquiterpene (E)-β-farnesene (EβF) is the major volatile alarm pheromone identified in most aphid species, including the pea aphid *Acyrthosiphon pisum*, the black bean aphid *Aphis fabae*, the green peach aphid *Myzus persicae*, and the grain aphid *Sitobion avenae*, while some species contain other alarm pheromone components [8,9]. For example, the vetch aphid *Megoura viciae* uses (–)-α-pinene, (–)-β-pinene, (+)-limonene, and EβF and their blends as alarm pheromones [10]. Aphid alarm pheromones play important roles in mediating aphid behaviors. It was reported that EβF had an obvious repellent activity against *S. avenae*, *Rhopalosiphum padi*, *A. pisum*, and *M. persicae* [2,11,12,13,14]; (–)-α-pinene, (–)-β-pinene, (+)-limonene, EβF, and their blends displayed strong, moderate, or weak repellency against *M. viciae* [10]. Nevertheless, the natural alarm pheromones are inherently unstable due to their conjugated double bonds. Thus, some analogs of aphid alarm pheromone have been designed and synthesized to enhance their stability, some of which demonstrated good repellent activity against *Schizaphis graminum*, *A. pisum*, or *M. persicae* [4,15,16,17,18,19].

Aphid alarm pheromone not only repels aphids, but it can also act as a kairomone to attract natural enemies. For instance, the EβF slow-release alginate formulation significantly attracted the parasitoid *Aphidius ervi* Haliday in crop fields [20]. EβF was also found to be the effective kairomone of the lady beetle *Adalia bipunctata* [21]. Behavioral experiments, electrophysiological recordings, and field trials showed that EβF, as a kairomone, could be detected by natural enemies [5,9,20,21,22,23,24]. A recent study reported that the larvae and adults of the aphid predator hoverfly *Eupeodes corollae* could detect and be attracted to EβF [5]. Although EβF as a kairomone of natural enemies has been well researched, other aphid alarm pheromone components have rarely been studied. In addition, it is still unclear whether aphid alarm pheromone analogs can act as kairomones to attract natural enemies.

*Diaeretiella rapae* McIntosh (Hymenoptera: Braconidae: Aphidiinae) is a widely used parasitoid against aphids [25]. It has been reported to parasitize more than 60 aphid species worldwide, such as *R. padi*, *M. persicae*, *Brevicoryne brassicae*, and *Lipaphis erysimi* [26,27]. *D. rapae* played an important role in suppressing aphid populations under natural field conditions, with a high parasitism rate of 15–70% in Brassica crops [27]. It was reported that the aphid alarm pheromone played a key role in attracting *D. rapae*. In the transgenic *Arabidopsis thaliana* plants of an EβF synthase gene, the emission of EβF elicited a potent response from *M. persicae* (alarm and repellent responses) and its parasitoid *D. rapae* (an arrestant response) [28], suggesting that EβF could act as a kairomone for *D. rapae*. Similarly, the transgenic tobacco plants of an EβF synthase gene emitted EβF at a high level—up to 19.25 ng/day—exhibiting enhanced repellence to *M. persicae* and attractiveness to *D. rapae* [29]. Some other studies also reported that *D. rapae* females were attracted to EβF [30,31]. Nevertheless, up to now, EβF is the only aphid alarm pheromone that has been tested against the natural enemies of aphids for its kairomonal activity. In the present study, we investigated the kairomonal effects of three monoterpene components, in addition to EβF and its analogs, using a two-way olfactometer on *D. rapae* females. Single compounds and blends were tested under three (low, medium, and high) concentrations.

## 2. Materials and Methods

### 2.1. Culture of Parasitoids

Parasitoid colonies of *D. rapae* were provided by the Key Laboratory for Agricultural Pest Management of the Mountainous Region (Prof. Wenlong Chen, Guizhou University, Guiyang 550025, China). They were reared on *M. persicae* maintained with seedlings of *Raphanus sativus* in the Laboratory of Insect Molecular Ecology at China Agricultural University (Beijing 100193, China) under conditions of 23 ± 1 °C, 60 ± 5% RH with a photoperiod of 16L: 8D in a climate incubator (RXZ-300B, Ningbo, China). *R. sativus* seedlings were planted in vermiculite, exposed to the aphids after germinating for 5 days, and replaced weekly to let the green peach aphids move to new seedlings independently.

### 2.2. Chemicals

Four aphid alarm pheromone components of (–)-α-pinene (purity 98%), (–)-β-pinene (purity 99%), (+)-limonene (purity 97%), and EβF (purity 95%) were purchased from Sigma-Aldrich Co., LLC. (St. Louis, MO, USA). *n*-Hexane of analytical purity grade was purchased from Sinopharm (Beijing, China). All other chemical reagents consisting of chemicals for the NMR, synthesis EβF analogs, and the solvent *n*-hexane were purchased from Shanghai Macklin Biochemical Co., Ltd. Silica gel (200–300 mesh, Puke Corporation, Qingdao, China) was used for column chromatographic purification.

### 2.3. Synthesis Procedure of EβF Analogs

**Figure 1:**
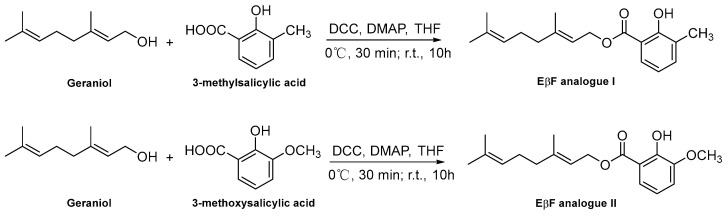
Scheme of the synthetic routes for EβF Analog I and II.

The target EβF analog I (E)-3,7-dimethylocta-2,6-dien-1-yl-2-hydroxy-3-methylbenzoate and EβF analog II (E)-3,7-dimethylocta-2,6-dien-1-yl-2-hydroxy-3-methoxybenzoate were synthesized via nucleophilic substitution reaction according to the reported method [4] (Figure 1). The synthesis of two EβF analogs was started by adding and stirring 3-methylsalicylic acid or 3-methoxysalicylic acid (10 mmol), dicyclohexylcarbodiimide (DCC, 12 mmol), and 4-dimethylaminopyridine (DMAP, 1 mmol) in anhydrous tetrahydrofuran (THF, 30 mL) for 0.5 h in an ice bath. The last reactant geraniol (10 mmol) was subsequently added dropwise and then stirred at room temperature for 10 h to yield a muddy white precipitation. After that, the produced mixture was cooled to 0 °C for 0.5 h, filtered, and the filtrate was then washed with water and chloroform three times. The filtered extract was washed with dichloromethane three times, and then the organic phases were combined, dried with anhydrous Na_2_SO_4_, filtered, concentrated, and purified with column chromatography on silica gel to yield EβF analogs I and II. The structures of the synthetic compounds were characterized by ^1^H NMR, ^13^C NMR and HRMS. The nuclear magnetic resonance spectra of two EβF analogs were determined on an AVANCE NEO spectrometer (Bruker, Bremen, Germany) at 500 MHz for ^1^H NMR and 125 MHz for ^13^C NMR, using tetramethylsilane (TMS) as an internal standard, and CDCl_3_ as the solvent. High resolution mass spectrometer (HRMS) data were obtained on a 7.0T FTICR-MS instrument (Varian, Palo Alto, CA, USA).

### 2.4. Olfactometer Bioassays

The samples including six single compounds ((–)-α-pinene, (–)-β-pinene, (+)-limonene, EβF, EβF analog I, and EβF analog II) and four blends (Blend I, Blend II, Blend III, and Blend IV, respectively) were diluted with *n*-hexane to three concentrations of 0.1, 1.0, and 5.0 μg/μL. Two blends, Blend I and Blend II (0.1, 1.0 and 5.0 μg/μL), were prepared in the following ratios: 1:44.4:6.5:2.2 and 1:18.4:1.3:0.8 ((–)-α-pinene, (–)-β-pinene, (+)-limonene, and EβF, respectively), which exhibited effective repellency against *M. viciae* [10]. A glass T-shaped two-way olfactometer (3.0 cm diameter, 10 cm trunk length, and 2.5 cm branch length) (Figure S1) was used to investigate the kairomonal effects of single alarm pheromone components, Blend I, Blend II, single EβF analogs and blends of EβF and its analog (Blend III: EβF + Analog I, Blend IV: EβF + Analog II, EβF:Analog = 1:1), on female parasitoids. In vivo olfactometry bioassays were performed following the method presented by Goelen et al. [32]. Forced air was dried and purified using an activated charcoal filter and bubbled through distilled water before flowing over the odor source and into each arm of the tube at a rate of 400 mL/min. Experiments were carried out in a dark room at 22 ± 1 °C with a lamp (24 W) placed centrally above the olfactometer at a height of 60 cm to provide uniform light. The parasitoids are very sensitive to light, and the behavioral response may be affected if light is not uniform. For assessing the parasitoid response to the alarm pheromone components and analogs, 10 μL of each sample was loaded on a filter paper. The solvent was allowed to evaporate for 30 s, and then the filter paper was placed in the stimulus source chamber of the olfactometer arm, while another filter paper was placed in the other chamber (10 μL of *n*-hexane was used as the control). All the samples—either the single component or blends of compounds—were freshly prepared to maintain accuracy in the olfactometer bioassays.

For each sample, 60 female parasitoids (12 groups of 5 individuals) were used [32]. Behavioral response was evaluated 10 min after the introduction of the parasitoids. A total of 1 group of 5 individuals served as a replicate, yielding a total of 12 replicates. The parasitoids moved freely towards any arm, and the number of individuals in each arm was recorded after 10 min. Parasitoids not making a choice within 10 min were deemed ‘non-responding’ and were not included in the statistical analysis. For each measurement, female individuals and filter papers were not reused. At the end of the test, the whole olfactometer was rinsed with distilled water, *n*-hexane, and finally ethanol, and air dried for the next use.

### 2.5. Behavioral Response Time Bioassay

Ten samples, (–)-α-pinene, (–)-β-pinene, (+)-limonene, EβF, Blend I, Blend II, single EβF analogs (Analog I and Analog II), and the blends (Blend III: EβF + Analog I, Blend IV: EβF + Analog II), were each dissolved in *n*-hexane to three concentrations of 0.1, 1.0, and 5.0 μg/μL. The behavioral response time of *D. rapae* females to each sample was evaluated using the same glass T-shaped two-way olfactometer. Under the same conditions as described in Section 2.4, one arm was filled with 10 μL of freshly prepared sample solution, and the other arm was filled with 10 μL of *n*-hexane as the control. For each experiment, 60 female parasitoids were individually released into the center of the two test arms. The time for each parasitoid to move 2.0 cm into the branch arm was recorded.

### 2.6. Statistical Analysis

Olfactometer bioassay responses of *D. rapae* to each sample were presented by calculating a preference index (PI), which represented the difference between the number of parasitoids choosing the samples and the solvent control divided by the total number of responding insects [32]: PI = (T − C)/(T + C) × 100%, where T and C represented the female parasitoid numbers in the treatment and control arms, respectively. In the olfactometer bioassay, the null hypothesis that displayed no bias for either olfactometer arm (i.e., 50:50 responses) was analyzed using a χ^2^ goodness-of-fit test to compare the number of individuals in the two olfactometer arms for each experiment [2], and as they were paired, comparisons with a control, *df* = 1, held for all. The number of parasitoids without a choice for either arm was not included in the data analysis. The PI values and behavioral response time were analyzed statistically using one-way analysis of variance (ANOVA), followed by Tukey’s B, test for the significant difference at *p* < 0.05. The presentation of the experiments, treatments, the numbers of parasitoids exposed to each, and the values were used for statistical analysis are summarized in Table S1 (Supplementary Materials). All data are presented as the mean ± SE (standard error). The software SPSS Statistics 21.0 (SPSS Inc., Chicago, IL, USA) was used in the statistical analysis.

## 3. Results

### 3.1. Synthesis of EβF Analogs

EβF Analog I and II were successfully synthesized by the esterification between the reactant geraniol and 3-methylsalicylic acid (or 3-methoxysalicylic acid) in anhydrous THF via the one-pot method at room temperature, and their structures were confirmed by ^1^H NMR, ^13^C NMR, and HRMS as analogs. Data for Analog I (File S1 and Figures S2–S4 in Supplementary Materials): yield: 32.5%; molecular weight: 288.39. Data for Analog II (File S2 and Figures S5–S7 in Supplementary Materials): yield: 55.2%; molecular weight: 304.39.

### 3.2. Olfactometer Bioassay: Response of D. rapae to Single Components of Alarm Pheromones

**Figure 2:**
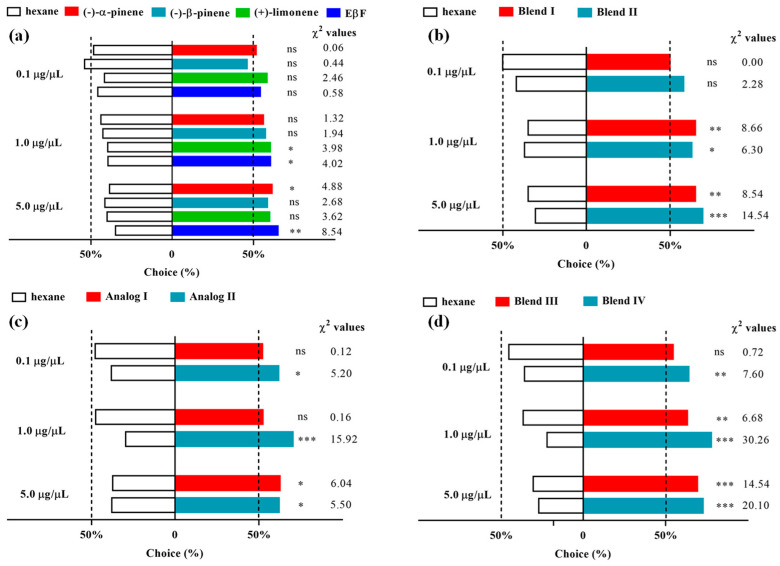
Behavioral response of *Diaeretiella rapae* to single and blend components of aphid alarm pheromone and analogs. (**a**) Response of *D. rapae* to single components of aphid alarm pheromone. (**b**) Response of *D. rapae* to blend components of aphid alarm pheromone. (**c**) Response of *D. rapae* to single components of analogs. (**d**) Response of *D. rapae* to blend components of EβF and analogs. (ns = no significant difference; *p* > 0.05; * *p* < 0.05; ** *p* < 0.01; *** *p* < 0.001).

Behavioral bioassay using single components of alarm pheromones showed that the response of *D. rapae* to all single components was weaker at lower concentrations (Figure 2a). Specifically, no single components elicited any significant response from *D. rapae* at the concentration of 0.1 μg/μL; EβF (*p* = 0.045), and (+)-limonene (*p* = 0.046) attracted significantly more parasitoids than the control group at 1.0 μg/μL; (–)-α-pinene (*p* = 0.0272) and EβF (*p* = 0.0035) attracted significantly and extremely significantly more parasitoids than the control group at 5.0 μg/μL, respectively; (–)-β-pinene showed no activity at all concentrations tested, and EβF was more attractive than each single compound. Similarly, when evaluated by the preference index (PI), *D. rapae* showed a weaker preference for all single components at a lower concentration, but its preference became stronger with increasing concentrations (Table 1). The PI values of all tested pheromone components, except (+)-limonene, were less than 7% at the concentrations of 0.1 μg/μL, but the PI values reached 18.9–33.6% when the concentration was increased to 5.0 μg/μL.

### 3.3. Olfactory Response of D. rapae to the Blend of Alarm Pheromones

Behavioral response tests showed that Blend I and Blend II elicited no significant response from *D. rapae* at the concentration of 0.1 μg/μL, but they elicited a significant and extremely significant response from *D. rapae* at 1.0 μg/μL and 5.0 μg/μL, respectively (Figure 2b). Specifically, at 1.0 μg/μL, Blend I attracted extremely significantly more parasitoids than the control group (*p* = 0.0033), and Blend II attracted significantly more parasitoids than the control (*p* = 0.0121). When evaluated by PI index, the parasitoids displayed a much stronger preference for higher concentrations (Table 1): the PI values at 0.1 μg/μL were less than 10%, while they were all over 30% at 1.0 μg/μL and 5.0 μg/μL. The PI value of Blend II reached 42.2% at 5.0 μg/μL.

### 3.4. Olfactory Response of D. rapae to Single EβF Analogs

Behavioral tests using EβF analogs with high aphid-repellent activity showed that Analog I and Analog II exhibited very different attractive activities to *D. rapae*. Analog I showed significant activity only at 5.0 μg/μL (*p* = 0.014), while Analog II exhibited significant or extremely significant activity at all concentrations tested (0.1 μg/μL: *p* = 0.0226; 1.0 μg/μL: *p* < 0.0001, and 5.0 μg/μL: *p* = 0.019) (Figure 2c). When evaluated by the PI index, Analog II also showed much higher PI values than Analog I at lower and medium concentrations, although they shared a similar PI value at higher concentrations (Table 1).

### 3.5. Olfactory Response of D. rapae to the Blend of EβF Analogs

Behavioral assays showed that EβF + Analog I (Blend III) was generally weaker than EβF + Analog II (Blend IV) in the attractive activity to *D. rapae* females at all concentrations tested (Figure 2d). Interestingly, the overall activity of the blends was enhanced compared to the single components. Both Blend III (0.1 μg/μL: *p* = 0.3961; 1.0 μg/μL: *p* = 0.0098; 5.0 μg/μL: *p* = 0.0001) and Blend IV (0.1 μg/μL: *p* = 0.0058; 1.0 μg/μL: *p* < 0.0001; 5.0 μg/μL: *p* < 0.0001) displayed distinct attractive activity to the parasitoids at 1.0 μg/μL and 5.0 μg/μL, and Blend IV showed significant activity at all concentrations tested. Meanwhile, the PI values of Blend IV were generally higher than those of Blend III, and the highest PI value was observed in Blend IV at 1.0 μg/μL (56.9%) (Table 1).

### 3.6. Two-Way Choice of D. rapae for Aphid Alarm Pheromones and Analogs

**Figure 3:**
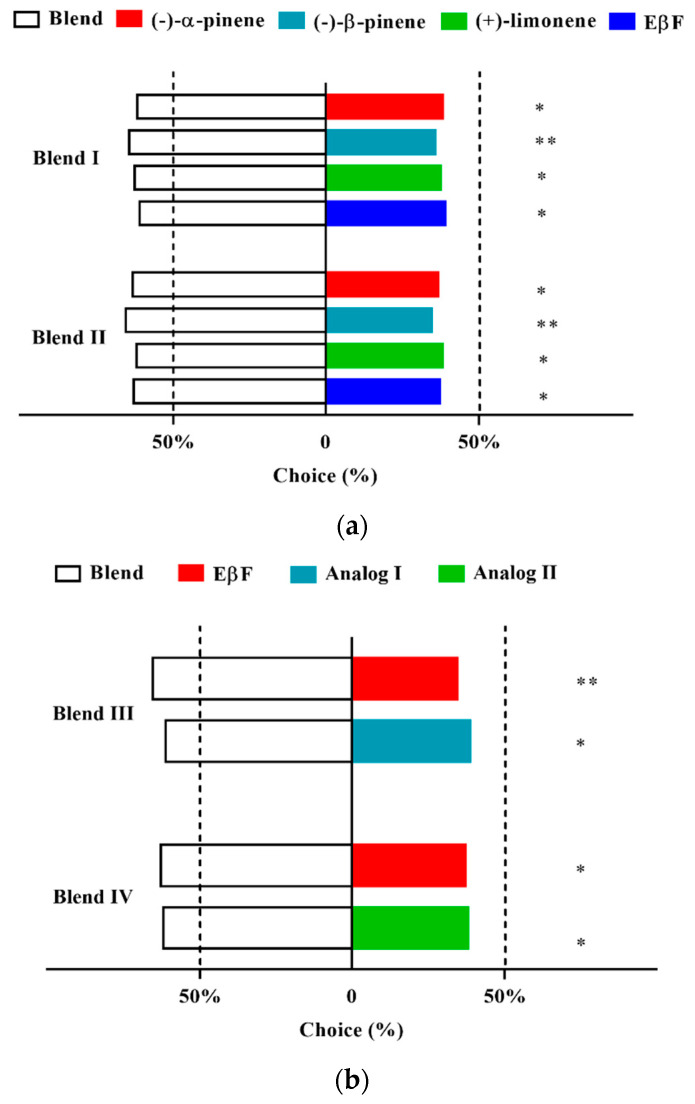
Two-way choice of *Diaeretiella rapae* between blends and single alarm pheromone components (**a**), and between blends and single compounds of EβF and its analogs (**b**). (* *p* < 0.05, ** *p* < 0.01).

In two-way choice experiments using aphid alarm pheromones, *D. rapae* preferred to choose Blend I and Blend II over each single alarm pheromone component, and Blend II was slightly more preferred by the parasitoids (Figure 3a). Similarly, in two-way choice experiments using EβF analogs and their blends, *D. rapae* preferred to choose the blends over each single compound. EβF + Analog I had similar attractive activity to EβF + Analog II (Figure 3b).

### 3.7. Olfactory Response Time of D. rapae to Aphid Alarm Pheromones and Analogs

**Figure 4:**
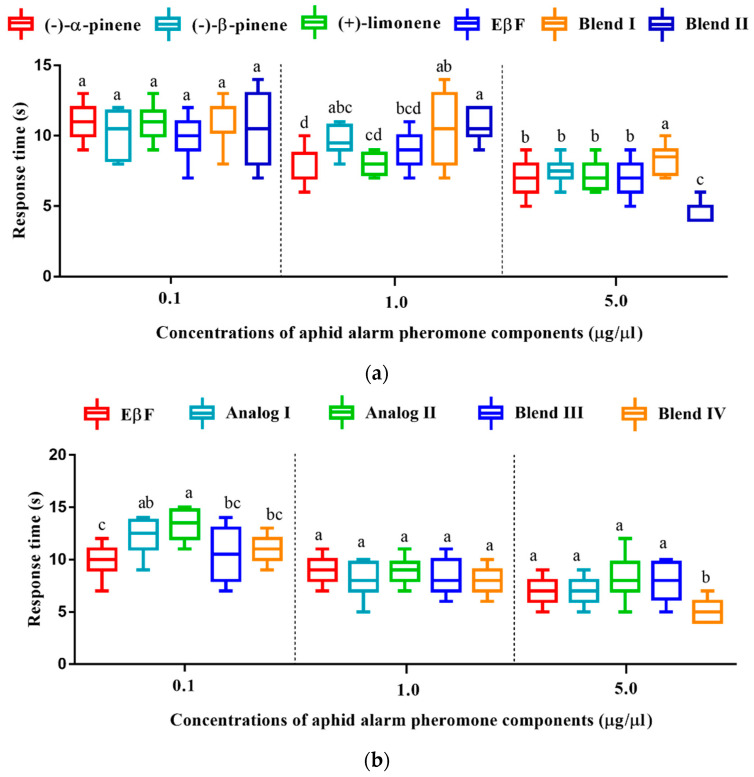
Mean (±SE) time, in seconds, for *Diaeretiella rapae* to choose single or blends of pheromone and analogs in a T-tube olfactometer. (**a**) Response time of *D. rapae* to single and blend components of aphid alarm pheromone. (**b**) Response time of *D. rapae* to single and blend compounds of EβF and its analogs. The significant differences in the response time of the compounds were analyzed statistically using one-way analysis of variance (ANOVA), followed by Tukey’s B test at *p* < 0.05. Different letters indicate significant differences.

The measurement of olfactory response time showed that the response time of *D. rapae* to alarm pheromone components and their blends shortened with increasing concentrations (Figure 4a). The parasitoids exhibited the longest response time at the concentration of 0.1 μg/μL, and there was no significant difference between different pheromone components and the blends. Interestingly, Blend I and Blend II yielded a conflicting response-time profile at different concentrations: they generally had the longest response time at 0.1 μg/μL and 1.0 μg/μL, but the response time of Blend II had the shortest response time at 5.0 μg/μL, while Blend I still had a longest response time at 5.0 μg/μL. When tested against EβF, the analogs, and their blends, once again, *D. rapae* exhibited a shortened response time with increasing concentrations (Figure 4b). There was no significant difference between EβF, the analogs, and their blends at 1.0 μg/μL. As expected, EβF + Analog II exhibited the shortest response time at 5.0 μg/μL, which showed the strongest attractive activity in the behavioral bioassay.

## 4. Discussion

Our results indicated that the blend of aphid alarm pheromone components was preferred over each single component by the parasitoid of aphid *D. rapae*. Moreover, the preference became stronger with increasing concentrations. The phenomenon that the parasitic wasps of aphid were attracted to higher concentrations of EβF has been observed in other studies. For example, the specialist parasitoid *Aphidius uzbekistanicus* (female) was attracted to higher concentrations of synthetic EβF (1.4 μg to 5.7 μg) [22]. The generalist parasitoids, *Aphidius ervi* and *Praon volucre*, were attracted to higher amounts of EβF (0.3–30.0 μg) [20,22,23,33]. Conversely, the responses of parasitic wasps to EβF can vary among different parasitoid species. For instance, the endoparasitoid of aphid *Aphidius colemani* consistently did not respond to EβF at any of the tested concentrations [34]. For other natural enemies of the aphid, the concentrations of EβF required to elicit a significant attractive response varied. The syrphid *E. corollae* could only be attracted by EβF when its amount was higher than 200.0 μg [5], but the syrphid *Episyrphus balteatus* could be attracted by as little as 5.0 μg of EβF [35]. In the present study, *D. rapae* females were significantly attracted by (+)-limonene at 1.0 μg/μL, but it was reported that the lacewing *C. pallens*, an important natural enemy of aphid, was not significantly attracted by 10.0 μg of limonene [36], although it could be attracted to (–)-α-pinene at an amount of 1.0 μg in a Y-tube olfactometer [36]. Our study showed that (–)-β-pinene elicited no significant response from *D. rapae* females at all concentrations tested, even though it could elicit electroantennogram responses from the aphids *Aphis fabae*, *S. avenea*, and *Metopolophium dirhodum* [37,38]. When the amount of (–)-β-pinene increased to 1000.0 μg, it attracted both sexes of the gravid ladybird *Harmonia axyridis* [39]. These studies suggest that the responses of natural enemies to aphid alarm pheromones are concentration-dependent. All previously reported studies tested only the activity of EβF to different natural enemies of aphids, but here we investigated the kairomonal effects of other aphid alarm pheromone components and their blends, in addition to EβF, on the parasitoid of the aphid, which contributes to our understanding of the mechanisms involved in regulation of the parasitoid behaviors of kairomones. Our study discovered a novel set of compounds that have a promising potential to be developed as kairomones for the biocontrol of aphids.

Our study also suggested that while individual aphid alarm pheromone components showed no significant attraction to the aphids’ natural enemies, the blends were very attractive. In this study, the single components (–)-α-pinene, (–)-β-pinene, (+)-limonene, and EβF showed no activity to *D. rapae* at specific concentrations, but when they were mixed at a ratio of 1:44.4:6.5:2.2 (Blend I) or 1:18.4:1.3:0.8 (Blend II), the blends showed significant activity at the concentration of 1.0 μg/μL or above. These results were verified by behavioral bioassay, two-way choice experiments, and the measurement of response time. We previously detected a significant repellent activity of Blend I and Blend II against the vetch aphid *M. viciae* [10], and here, for the first time, we detected a significant kairomonal activity of Blend I and Blend II to the parasitoid of aphid *D. rapae*. It is not rare that an insect’s natural enemies are attracted to the pheromone blend over individual components. For instance, the blend of geranyl acetone, EβF, β-citronellol, β-caryophyllene, α-humulene, β-pinene, β-citronellal, limonene, anisaldehyde, and citral displayed a higher attractive activity, compared with any single compound, to most hoverflies in forest habitats [40]. The predatory bug *Orius laevigatus* was only attracted to the blend of (R)-lavandulyl acetate and neryl (S)-2-methylbutanoate at a ratio of 1:2.3, the major aggregation pheromone components of its prey *Frankliniella occidentalis* [41,42]. Field data also showed that, compared with single volatiles, the mixture of plant volatiles methyl salicylate, (Z)-3-hexenol, and (Z)-3-hexenyl acetate attracted more *Stethorus punctum picipes*, an obligate ladybeetle predator of tetranychid mites [43]. Nevertheless, there were also reports that single volatiles were more attractive than blends. For example, the syrphid flies *E. volucris* and *E. fumipennis* preferred 2-phenylethanol over the blend of acetic acid, acetophenone, and 2-phenylethanol [44]. Generally, the blends of compounds can be more attractive than single components.

Our most important finding in this study is that the blend of EβF and the analogs elicited a stronger response from *D. rapae* than individual analogs and all aphid alarm pheromone components tested here. Synthetic EβF analogs have been previously reported to have alarm and repellent activities [4,15,16,17,18,19], but their kairomonal activity have not been reported. EβF Analog I and Analog II tested in this study were previously shown to have high repellent activity against aphids [4]. The olfactory response bioassay indicated that both Analog I and Analog II were attractive to *D. rapae*, although Analog II was more attractive than Analog I at lower concentrations. The attractive activity of Analog II was also significantly higher than that of EβF at lower concentrations. When mixing EβF with either Analog I or Analog II, the attractive activity of the blends was substantially enhanced. The highest activity was recorded in the EβF + Analog II at 1.0 μg/μL and 5.0 μg/μL. It displayed a stronger activity than any other compounds tested in the present study. Our data provided a promising opportunity for developing kairomones for the natural enemies of aphids.

## 5. Conclusions

The blend of aphid alarm pheromone components is more preferred by the parasitoid *D. rapae* than are the individual components. While individual pheromone components did not show kairomonal activity at lower concentrations, blends of these components were attractive. Our study showed, for the first time, a strong kairomonal effect using a blend of aphid alarm pheromone components and their analogs over any single component alone. All previous studies tested only the activity of EβF on aphids’ natural enemies, but here we confirmed the kairomonal effect of other aphid alarm pheromone components and their blends in addition to EβF on the parasitoid of aphids, which provided a potential factor in the behavior of parasitoids in relation to their prey. Our study provides a promising opportunity for designing kairomones to attract the natural enemies of aphids.

## Figures and Tables

**Table 1:** Mean (±SE) preference index (PI *) for *D. rapae* to aphid alarm pheromone components and analogs.

Compounds	Concentrations (µg/µL)		
	0.1	1.0	5.0
(–)-α-pinene	5.6 ± 4.8 lmn	10.0 ± 4.4 i–n	21.4 ± 2.9 e–i
(–)-β-pinene	1.1 ± 1.9 n	14.7 ± 1.3 h–n	18.9 ± 4.8 g–m
(+)-limonene	16.9 ± 7.0 h–n	21.1 ± 3.4 e–i	19.2 ± 9.5 f–m
EβF	6.1 ± 1.9 k–n	21.9 ± 1.3 e–k	33.6 ± 0.5 b–g
Analog I	4.4 ± 4.2 mn	4.4 ± 1.0 mn	25.8 ± 3.8 d–i
Analog II	25.0 ± 3.0 d–i	39.4 ± 2.5 bcd	23.1 ± 2.4 e–j
Blend I	9.7 ± 5.5 i–n	34.2 ± 6.5 b–g	36.7 ± 6.0 bcde
Blend II	6.9 ± 8.7 j–n	30.8 ± 4.3 c–h	42.2 ± 4.7 bc
Blend III	8.3 ± 3.8 j–n	35.3 ± 13.5 b–f	40.8 ± 3.0 bcd
Blend IV	25.6 ± 7.9 d–i	56.9 ± 1.7 a	47.8 ± 9.6 ab

*: The preference index (PI) of each compound was calculated by the formula PI = (T − C/(T + C) × 100%, where T and C represented the female parasitoid numbers in the treatment and control arms, respectively. Values are mean ± standard error. The significant differences in the PI values of the compounds were analyzed statistically using one-way analysis of variance (ANOVA), followed by Tukey’s B test at *p* < 0.05. Different letters indicate significant differences.

## Data Availability

All data generated or analyzed during this study are included in this article and its Supplementary Information Files.

## Supplementary Materials

The following supporting information can be downloaded at: https://www.mdpi.com/article/10.3390/insects13111055/s1, Table S1: The sets for olfactometer bioassays and behavioral response time bioassay; File S1: Structural characterization data for EβF Analog I; File S2: Structural characterization data for EβF Analog II; Figure S1: Olfactometer device for bioassay of behavioral response of *Diaeretiella rapae* to components and analogs of aphid alarm pheromone; Figure S2: ^1^H NMR spectra of EβF Analog I; Figure S3: ^13^C NMR spectra of EβF Analog I; Figure S4: HRMS spectra of EβF Analog I; Figure S5: ^1^H NMR spectra of EβF Analog II; Figure S6: ^13^C NMR spectra of EβF Analog II; Figure S7: HRMS spectra of EβF Analog II.
