# Supplementary material for: Kairomonal Effect of Aphid Alarm Pheromones and Analogs on the Parasitoid Diaeretiella rapae
Source: Insects. 2022 Nov 15;13(11):1055. doi: 10.3390/insects13111055 (PMC9697894; doi:10.3390/insects13111055)
Supplement: Supplementary file 1 [file insects-13-01055-s001.zip › insects-2019706-supplementary.pdf]

Kairomonal Effect of Aphid Alarm Pheromones and Analogs on the Parasitoid *Diaeretiella rapae*

Yaoguo Qin *, Shangyang Zhang and Zhengxi Li *

Department of Entomology and MOA Key Laboratory for Monitoring and Environment-Friendly Control of Crop Pests, College of Plant Protection, China Agricultural University, Beijing 100193, China

* Correspondence: qinyg1018@163.com (Y.Q.); zxli@cau.edu.cn (Z.L.)

Supplementary

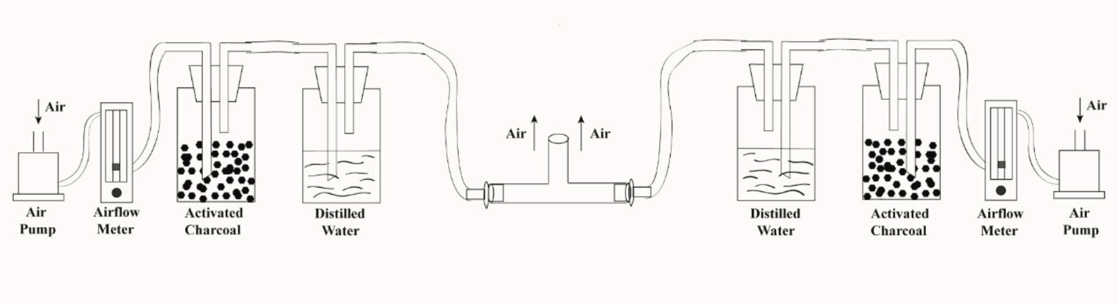

Figure S1. Olfactometer device for bioassay of behavioral response of *Diaeretiella rapae* to components and analogs of aphid alarm pheromone.

Table S1. The sets for olfactometer bioassays and behavioral response time bioassay

treatments		concentrations	numbers of tested parasitoids	values for statistical analysis
Single components	(-)-α-pinene	0.1μg/μL	60 females	(Choice percentage) comparing the number of individuals in the control and treatment arms: χ^2 goodness-of-fit test
	(-)-β-pinene	1.0 μg/μL		
	(+)-limonene	5.0 μg/μL		
	EβF			
	Analog I			
	Analog II			
Blends	Blend I			PI values and response times: ANOVA followed by Tukey's B test at P<0.05
	Blend II			
	Blend III			
	Blend IV			

File S1. Structural characterization data for EβF Analog I

^{1}H NMR (500 MHz, $CDCl_3$): δ ppm 11.10 (s, 1H, ArOH), 7.70 (dd, 1H, J_1 = 8.00 Hz, J_2 = 1.15 Hz, ArH), 7.30 (d, 1H, J = 7.25 Hz, ArH), 6.77 (t, 1H, J = 3.85 Hz, ArH), 5.44-5.47 (m, 1H, C=CH), 5.07-5.10 (m, 1H, C=CH), 4.85 (d, 2H, J = 7.10 Hz, CH_2-O), 2.26 (s, 3H, Ar-CH_3), 2.07-2.15 (m, 4H, C-CH_2CH_2-C), 1.77 (s, 3H, C-CH_3), 1.68 (s, 3H, C-CH_3), 1.60 (s, 3H, C-CH_3); ^{13}C NMR (125 MHz, $CDCl_3$): 170.64, 160.06, 143.18, 136.31, 131.96, 127.51, 126.54, 123.66, 118.38, 117.81, 111.97, 62.13, 39.55, 26.27,

25.58, 17.72, 16.60, 15.70. HRMS (ESI$^-$) *m/z* calcd for $C_{18}H_{23}O_3$ [M-H]$^-$ 287.16417; found: 287.16443.

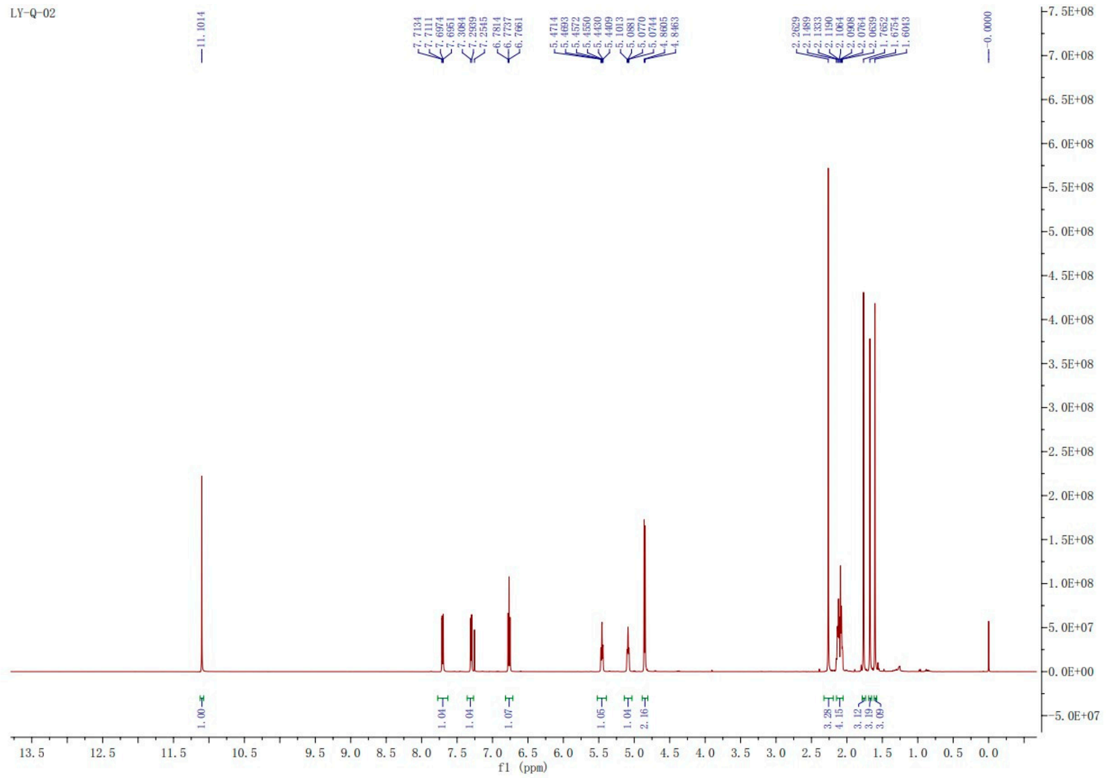

Figure S2. ^{1}H NMR spectra of EβF Analog I

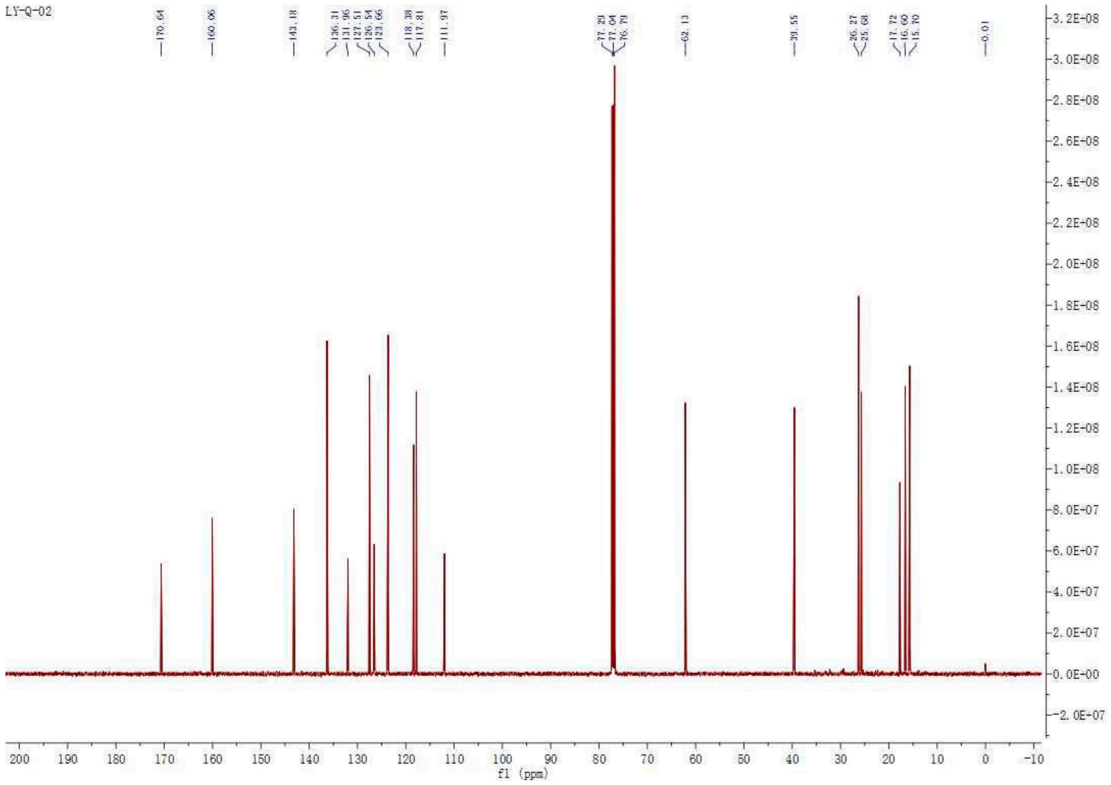

Figure S3. ^{13}C NMR spectra of EβF Analog I

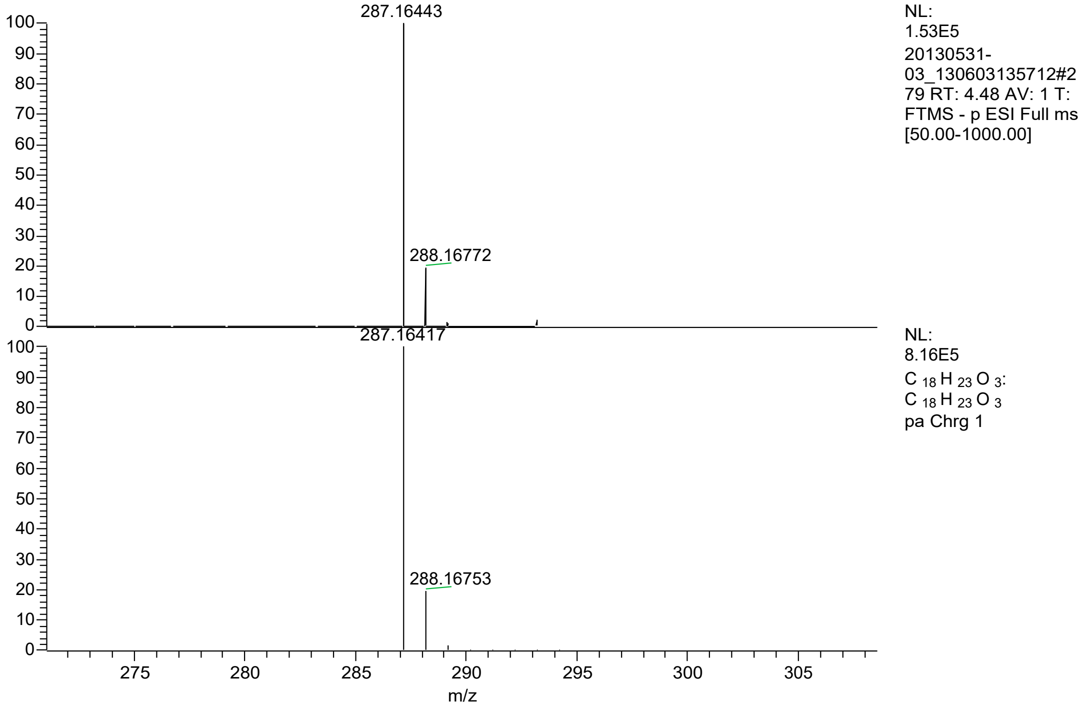

Figure S4. HRMS spectra of EβF Analog I

File S2. Structural characterization data for EβF Analog II

^{1}H NMR (500 MHz, $CDCl_3$): δ ppm 11.11 (s, 1H, ArOH), 7.45-7.46 (dd, 1H, J_1 = 8.15 Hz, J_2 = 1.45 Hz, ArH), 7.03 (d, 1H, *J* = 7.25 Hz, ArH), 6.82 (t, 1H, *J* = 8.05 Hz, ArH), 5.44-5.47 (m, 1H, C=CH), 5.07-5.10 (m, 1H, C=CH), 4.87 (d, 2H, *J* = 7.10 Hz, CH_2-O), 3.90 (s, 3H, O-CH_3), 2.07-2.15 (m, 4H, C-CH_2CH_2-C), 1.77 (s, 3H, C-CH_3), 1.68 (s, 3H, C-CH_3), 1.60 (s, 3H, C-CH_3). ^{13}C NMR (125 MHz, $CDCl_3$): 170.46, 152.08, 148.52, 143.37, 131.97, 123.62, 121.18, 118.39, 117.66, 116.40, 112.94, 62.32, 56.18, 39.55, 26.25, 25.68, 17.72, 16.61. HRMS (ESI^-) *m/z* calcd for $C_{18}H_{23}O_4$ $[M-H]^-$ 303.15909; found: 303.15921.

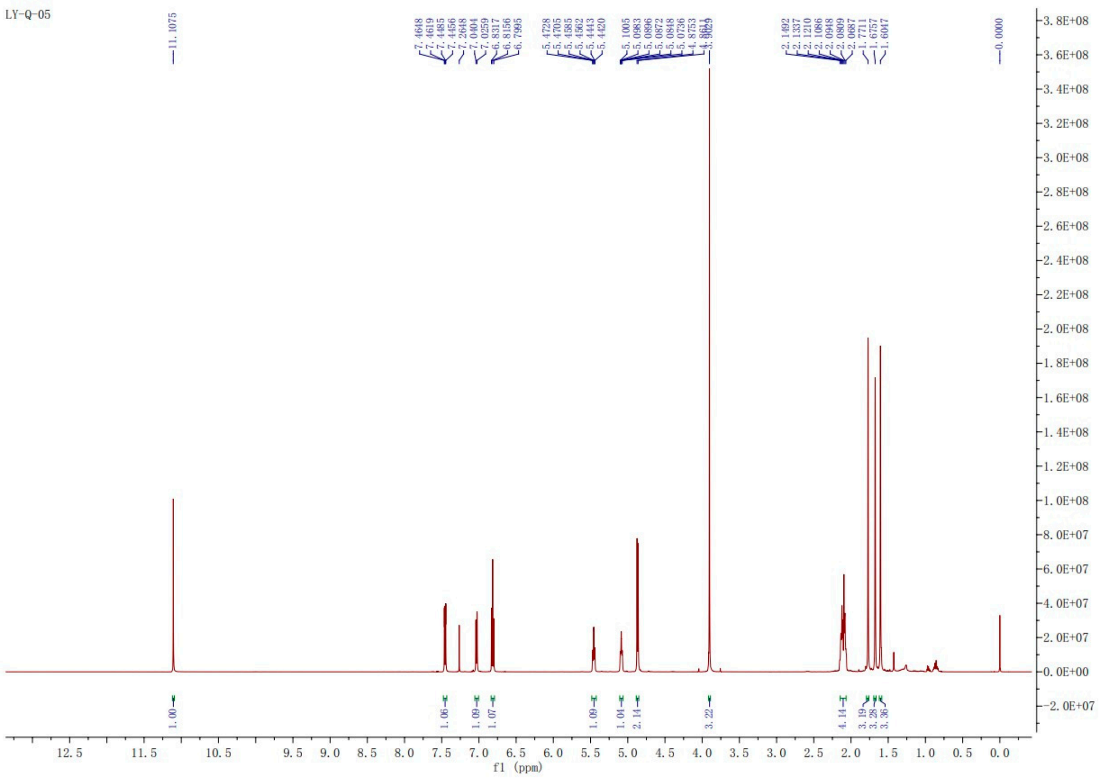

Figure S5. ^{1}H NMR spectra of EβF Analog II

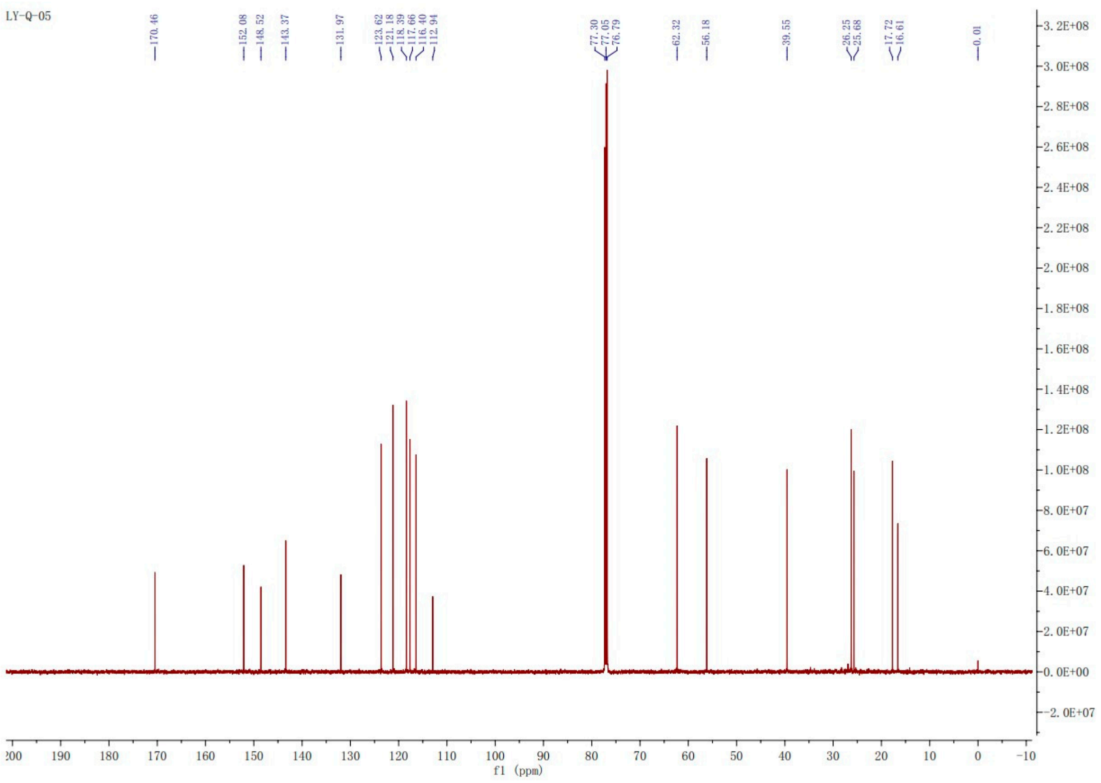

Figure S6. ^{13}C NMR spectra of EβF Analog II

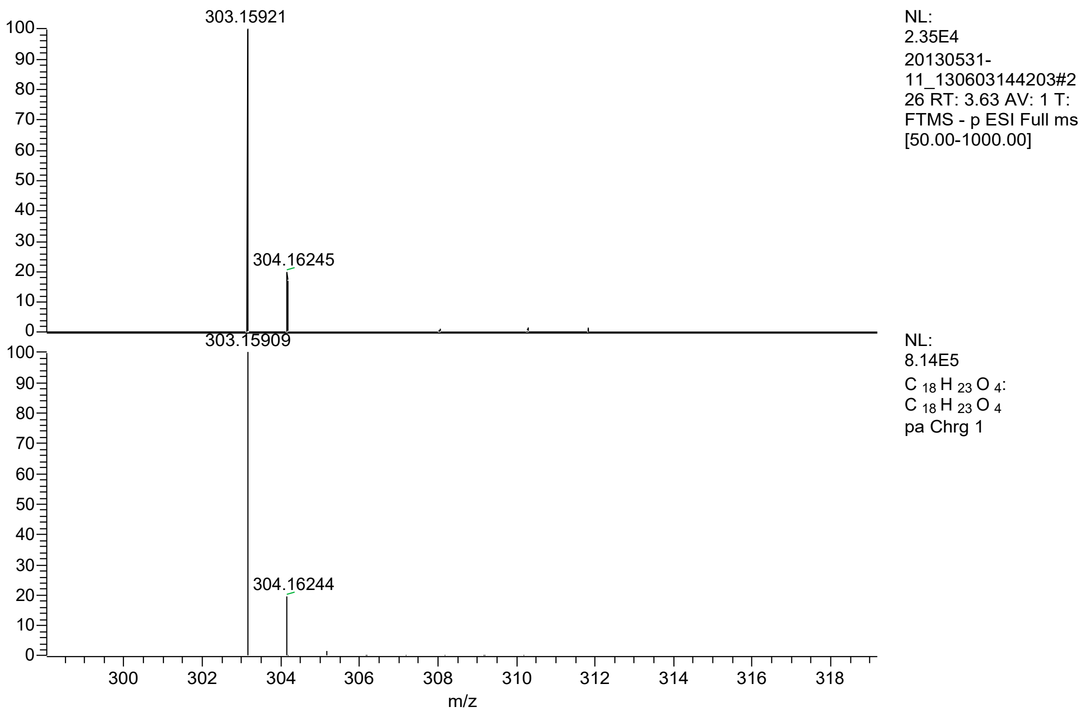

Figure S7. HRMS spectra of EβF Analog II
